# Plasma cell-free DNA PCR for diagnosing mucormycosis: are we there yet?

**DOI:** 10.1128/jcm.01350-25

**Published:** 2025-10-09

**Authors:** Valliappan Muthu, Inderpaul Singh Sehgal, Ritesh Agarwal

**Affiliations:** 1 Department of Pulmonary Medicine, Postgraduate Institute of Medical Education and Research 685015 , Chandigarh, India; University of Utah, Salt Lake City, Utah, USA

**Keywords:** pulmonary mucormycosis, zygomycosis, PCR, mucorales

## LETTER

We read with interest the study by Mau et al. evaluating cell-free DNA (cfDNA) polymerase chain reaction (PCR) for mucormycosis, which reported promising diagnostic performance (sensitivity 85%, specificity 93%), with notable differences between immunosuppressed (92%) and non-immunosuppressed (55%) patients.(1) While cfDNA represents an important diagnostic frontier, several critical methodological concerns limit the interpretability of these findings.

### Retrospective design introduces systematic bias

The retrospective case-control design compromises diagnostic accuracy estimates. Contrary to the authors’ assertion that prospective studies might yield higher sensitivity, available evidence demonstrates the opposite pattern.(2–4) Our recent meta-analysis of 23 studies revealed substantially lower pooled sensitivity in prospective versus retrospective designs (47% vs 64%).(5) Retrospective designs introduce selection bias by including only patients with known outcomes, artificially inflating performance metrics. The absence of blinding further compounds this issue, as knowledge of test results influenced clinical management, creating a circular validation process deviating from fundamental diagnostic principles.(1)

### Incorporation bias undermines diagnostic validity

Including cfDNA PCR results within the reference standard for probable mucormycosis (modified European Organization for Research and Treatment of Cancer and the Mycoses Study Group Education and Research Consortium [EORTC/MSGERC] criteria) violates diagnostic test evaluation principles. This incorporation bias produces a tautological scenario where the test partly defines the condition it purports to detect. Its impact is evident from the higher sensitivity in probable cases (92%) compared with proven cases (79%), where the standard remained independent of the index test.(1) While acknowledged as a limitation, this flaw critically undermines the study’s conclusions and contravenes STARD reporting guidelines.(6) Performance claims presented in the abstract therefore risk being misleading.

### Multiple testing inflates sensitivity estimates

The study provides no information on the number of samples tested per patient, a crucial determinant of sensitivity. Many cfDNA studies reporting high performance use repeated (often biweekly) screening, where any positive result classifies the patient as positive, fundamentally altering test characteristics. One prospective study demonstrating 85% sensitivity reported a median of seven samples in PCR-positive versus two in negative patients.(7, 8) Contrarily, a recent prospective study in patients with diabetes and pulmonary mucormycosis found single-timepoint plasma PCR sensitivity to be only 19% (specificity 91%).(2) Our meta-analysis demonstrated a 14% sensitivity increment with each additional blood sample tested.(5) Without standardized single-timepoint testing, sensitivity estimates become incomparable across studies and do not reflect real-world diagnostic scenarios.

### Analytical reproducibility concerns

Concerns also remain regarding inter- and intra-laboratory reproducibility. Although a French multicenter data suggested consistency (7), another French study reported only 52% reproducibility upon retesting.(9) Moreover, the variation between commercial and in-house PCR platforms limits the generalizability of findings across laboratories.(5, 10)

### Path forward: standardized prospective evaluation

While cfDNA may eventually prove valuable in high-risk populations, its diagnostic utility remains unproven under rigorous methodological conditions. Future studies should prioritize:

1. **Prospective, blinded study designs** with predetermined testing protocols
2. **Independent reference standards** excluding the index test
3. **Single-time-testing** protocols that reflect clinical decision-making scenarios
4. **Standardized analytical platforms** with demonstrated inter-laboratory reproducibility
5. **Clear distinction** between screening and diagnostic applications

Until such studies are conducted, routine clinical implementation of cfDNA PCR for mucormycosis diagnosis, particularly single-timepoint testing in non-immunosuppressed individuals, remains premature and unsupported by current evidence.

## EDITOR'S NOTE

Ed. Note: The authors of the original article did not respond at the time of publication.

## References

[1] Mah J, Lieu A, Nicholas V, Moreno A, Murugesan K, Budvytiene I, Banaei N. 2025. Accuracy of plasma cell-free DNA PCR for non-invasive diagnosis of mucormycosis. J Clin Microbiol 63:e0079625. doi:10.1128/jcm.00796-25 40631916 PMC12345248

[2] Nawaz RS, Agarwal R, Rudramurthy SM, Choudhary H, Harchand R, Kumar K, Sehgal IS, Kaur H, Dhooria S, Prasad KT, Prabhakar N, Aggarwal AN, Muthu V. 2025. Sensitivity and specificity of plasma and bronchoalveolar lavage fluid PCR for diagnosing pulmonary mucormycosis in subjects with diabetes mellitus. Mycoses 68:e70063. doi:10.1111/myc.70063 40257000

[3] Seo H, Kim JY, Son HJ, Jung J, Kim MJ, Chong YP, Lee SO, Choi SH, Kim YS, Kim SH. 2021. Diagnostic performance of real-time polymerase chain reaction assay on blood for invasive aspergillosis and mucormycosis. Mycoses 64:1554–1562. doi:10.1111/myc.13319 34013523

[4] Pandey M, Xess I, Sachdev J, Sharad N, Gupta S, Singh G, Yadav RK, Rana B, Raj S, Ahmad MN, Nityadarshini N, Baitha U, Soneja M, Prakash B, Sikka K, Mathur P, Jyotsna VP, Kumar R, Wig N, Gourav S, Biswas A, Thakar A. 2024. Utility of an in-house real-time PCR in whole blood samples as a minimally invasive method for early and accurate diagnosis of invasive mould infections. J Infect 88:106147. doi:10.1016/j.jinf.2024.106147 38555035

[5] Muthu V, Agarwal R, Dhooria S, Sehgal IS, Prasad KT, Rudramurthy SM, Aggarwal AN, Chakrabarti A. 2025. Sensitivity and specificity of polymerase chain reaction in blood and bronchoalveolar lavage samples for mucormycosis: a Bayesian diagnostic test accuracy meta-analysis. Mycopathologia 190:88. doi:10.1007/s11046-025-00996-w 40963061

[6] Bossuyt PM, Reitsma JB, Bruns DE, Gatsonis CA, Glasziou PP, Irwig L, Lijmer JG, Moher D, Rennie D, de Vet HCW, Kressel HY, Rifai N, Golub RM, Altman DG, Hooft L, Korevaar DA, Cohen JF, STARD Group. 2015. STARD 2015: an updated list of essential items for reporting diagnostic accuracy studies. Radiology 277:826–832. doi:10.1148/radiol.2015151516 26509226

[7] Millon L, Caillot D, Berceanu A, Bretagne S, Lanternier F, Morio F, Letscher-Bru V, Dalle F, Denis B, Alanio A, Boutoille D, Bougnoux ME, Botterel F, Chouaki T, Charbonnier A, Ader F, Dupont D, Bellanger AP, Rocchi S, Scherer E, Gbaguidi-Haore H, Herbrecht R. 2022. Evaluation of serum mucorales polymerase chain reaction (PCR) for the diagnosis of mucormycoses: the MODIMUCOR prospective trial. Clin Infect Dis 75:777–785. doi:10.1093/cid/ciab1066 34986227

[8] Rudramurthy SM, Muthu V, Agarwal R. 2024. Contemporary diagnosis and epidemiological trends of mucormycosis: a call for action and caution. Lancet Reg Health Eur 45:101039. doi:10.1016/j.lanepe.2024.101039 39262449 PMC11387205

[9] Ghelfenstein-Ferreira T, Verdurme L, Alanio A. 2022. Analytical performance of the commercial mucorgenius assay as compared to an in-house qPCR assay to detect mucorales DNA in serum specimens. J Fungi (Basel) 8:786. doi:10.3390/jof8080786 36012775 PMC9410016

[10] Rafanomezantsoa LC, Sabourin E, Guennouni Sebbouh N, Sitterlé E, Ben Halima N, Raveloarisaona YS, Quesne G, Dannaoui E, Bougnoux M-E. 2024. Agreement between two real-time commercial PCR kits and an in-house real-time PCR for diagnosis of mucormycosis. Microbiol Spectr 12:e0358523. doi:10.1128/spectrum.03585-23 38916337 PMC11302037

